# Exploring Co-production as an Implementation Strategy for Trauma-Informed Care in a Youth-Focused HIV Clinic in Memphis, Tennessee: Mixed Methods Research

**DOI:** 10.2196/66426

**Published:** 2025-08-21

**Authors:** Leslie Lauren Brown, Megan L Wilkins, Robert Kenneth Douglas McLean, Jamie Lynn Stewart, Nehali D Patel, Almariana Jessica Acuña, Carolyn Marie Audet, Jessica M Sales, April C Pettit, Latrice C Pichon

**Affiliations:** 1 Family and Community Medicine, Psychiatry and Behavioral Sciences, School of Medicine, Meharry Medical College, 1005 Dr. D.B. Todd Jr. Boulevard, Nashville, TN, 37208, United States, 1 6155573499; 2 Department of Infectious Diseases, St. Jude Children's Research Hospital, Memphis, TN, United States; 3 The Centre for Practice-Changing Research, Ottawa Hospital Research Institute, Ottawa, ON, Canada; 4 Department of Health Policy and the Institute for Global Health, Vanderbilt University Medical Center, Nashville, TN, United States; 5 Rollins School of Public Health, Emory University, Atlanta, GA, United States; 6 Infectious Disease Division, School of Medicine, Vanderbilt University Medical Center, Nashville, TN, United States; 7 School of Public Health, University of Memphis, Memphis, TN, United States

**Keywords:** HIV, trauma-informed care, co-production, youth, research quality

## Abstract

**Background:**

Memphis, Tennessee is second in the nation for HIV incidence, with one in three diagnoses among youth. Psychological trauma disproportionately impacts youth with HIV, compared with HIV-negative counterparts, requiring community-led and trauma-informed solutions to address mental wellness among youth with HIV. However, a dearth of research concentrates on trauma-informed care (TIC) for this population, with little exploration among youth-centered HIV care settings or into strategies for mobilizing communities to develop solutions. Research co-production*,* an approach in which research beneficiaries engage in research as cooperative partners, aligns with the TIC focus on collaborative decision-making and could be an effective strategy for facilitating collaborative TIC adoption, but formative research is needed to explore this potential.

**Objective:**

We sought to explore TIC implementation determinants and contextual factors that might influence research co-production as a strategy for implementation, including appetite for evidence-based approaches, support for co-production, and resources for capacity building.

**Methods:**

We applied an exploratory sequential mixed methods design to identify potential barriers and facilitators to TIC implementation in a youth-focused clinic and contextual factors relative to co-production. All clinic personnel were purposively invited to complete semistructured interviews. Thematic analysis, via four cycles of coding, was applied using the Consolidated Framework for Implementation Research 2.0 to qualitative data. Subsequently, a steering committee of clinic personnel was invited to complete surveys, applying the Research Quality Plus for Co-Production framework to explore co-production factors. A deliberative dialog approach was applied to analyze these findings and synthesize them with Consolidated Framework for Implementation Research.

**Results:**

A total of 20 personnel completed interviews, and 9 completed surveys. Potential facilitators included perceived clinic cohesiveness, equity focus, and prioritization or compatibility of TIC. Potential barriers included perceived disconnect between the clinic and larger hospital (in which youth with HIV were seen as stigmatized in other areas of the hospital), sustainability concerns related to a perceived lack of championing by leaders, insufficient mental health protocols, a lack of formal patient feedback procedures, and a lack of protected time for personnel activity engagement. Survey responses suggested that the clinic is likely supportive of evidence-based approaches (mean 3.6, SD 0.70) and collaborative research (mean 3.1, SD 0.31) and empowers personnel to participate (mean 3.1, SD 0.22). Conducive to co-production, the environment was seen as learning-centered, where evidence and standardized or validated approaches are prioritized, and there is an openness for innovation, with a focus on health disparities and quality improvement. Potential barriers included change-resistant staff, role silos, and underutilization of staff skills, coupled with a lack of formal research training and time constraints.

**Conclusions:**

Findings suggested that TIC implementation is likely to be embraced in the clinic, with co-production perceived as useful and fitting. However, greater effort is needed to integrate patient experiences and test co-production as a TIC implementation strategy.

## Introduction

Memphis, Tennessee, has seen a worsening HIV epidemic and is now second in the nation for incidence [1,2]. Youth aged 13 to 24 years in the area account for one in three people newly diagnosed with HIV [3], representing a 40% increase in HIV incidence between 2018 and 2023 [4]. Psychological trauma is a modifiable but understudied driver of poor health outcomes among youth with HIV, having been connected with care attrition, poor medication adherence, and unsuppressed viremia [5-9]. As youth with HIV in Tennessee experience disproportionately higher rates of posttraumatic stress disorder than HIV-negative counterparts (41% vs 2%, respectively) [5,10,11], local plans to end the HIV epidemic in Memphis and other priority jurisdictions identify implementation of trauma-informed care (TIC) as critical for improving HIV outcomes [12,13]. Yet, strategies for implementing TIC effectively in youth-focused HIV care clinics remain understudied. Meanwhile, HIV governing bodies urge that the HIV epidemic will not be halted without community-led solutions to attenuate health disparities [2,14].

A key feature of TIC is the belief that factors related to culture, history, and gender influence health outcomes, and intentional efforts are needed to acknowledge and minimize power differentials as part of decision-making processes [15]. As an evidence-based approach for preparing personnel to realize, recognize, and respond to trauma to resist retraumatization [16], TIC is associated with multilevel improvements. At the personnel level, it has been connected to improvements in organizational culture and professional quality of life [17], and at the patient level, it has the potential to improve health outcomes among youth [18] and adults with HIV [19]. In Tennessee, a trauma intervention was associated with a 15-times increase in retention in HIV care and a 5-times increase in viral suppression [20]. Although TIC is a burgeoning approach to care, strategies for trauma-informed research appear less frequently [21]. Trauma-informed research focuses attention toward fostering safe research activities, so that all involved in research recognize potential signs of trauma-related distress and protocols are in place to minimize distress [21]. For example, TIC principles call for efforts to promote collaborative decision-making and mutuality while considering historical contexts [2]. Despite the potential for TIC to improve health outcomes, replicable implementation of TIC presents as challenging, as it relies on flexible principles that vary by setting as to how they can or should be applied [22].

Research co-production is an umbrella term for many approaches to creating knowledge where persons who will benefit from the research (ie, knowledge users and beneficiaries)—such as people with HIV or those with experience providing care in HIV care organizations—are engaged in the research process as cooperative partners [23]. Collaborative research efforts, in which research knowledge users or beneficiaries are engaged as equal decision makers, are therefore well-positioned to ensure solutions are rooted in lived experience, experiential understandings of preferences for care, and viable research strategies. Research conducted without such feedback is more likely to be wasteful, less effective [24], and carries potential to propagate harm by reenacting traumatic power dynamics [25], including the objectification, minimization, and dismissal of voices, perspectives, and priorities of people with little or no political or financial power [22] or those considered to lack “scientific expertise.” [23]

Co-production is highly compatible with TIC, as both emphasize the importance of collaboration, the empowerment and elevation of the voices of persons with lived experience, and attention to minimizing power differentials [15]. However, to our knowledge, no frameworks exist to guide collaborative TIC implementation via research co-production. This knowledge and practice gap poses challenges for developing community-led solutions in HIV care organizations, as scant literature presents findings relative to the usefulness or potential for co-production as a strategy for implementing TIC. Overall, limited literature explores the utility of collaborative research as part of TIC implementation [15]. Specifically, gaps remain relative to how to replicate TIC, indicating a need to develop and test reproducible strategies and indications for how they might be culturally adapted across settings. Further, little information exists about how HIV care personnel perceive the potential usefulness of adopting a co-production approach.

There is an urgent need for community-led approaches to attenuate trauma effects in adolescent HIV clinics in the Southern United States. However, formative research is needed to first understand how personnel working in these care spaces view TIC and co-production. Thus, the primary objective of this study was to conduct exploratory research to guide the preliminary development of a collaborative TIC implementation plan in a youth-focused HIV clinic. We sought to explore personnel-perceived barriers and facilitators to TIC implementation in the setting, as well as perception of current research practices that might influence adoption of a co-production strategy. Specifically, we sought to understand how personnel perceived the research milieu relative to knowledge use environment, evidence as a priority, and the overall application of collaborative research. By answering these research questions, we aim to apply findings to develop a replicable approach for guiding collaborative TIC implementation with and for people with lived experience who have been most affected by trauma.

## Methods

### Study Setting

This study focuses primarily on the experiences of personnel from an adolescent and young adult-focused HIV clinic in the priority jurisdiction of Memphis, Tennessee. This clinic, serving approximately 200 patients, is housed within a larger tertiary pediatric hospital, with a primary focus on hematology and oncology. The clinic is the primary provider for HIV care for youth aged 14‐24 years in a tristate region. Patients are primarily racial minorities, with about 70% represented by cisgender men who have sex with men and the remaining cisgender women. Patients of this clinic have access to institutional transportation, meals, research activities, school advocacy, mental health services, and case management via social workers, a psychologist, a psychiatrist, and a chaplain, sexually transmitted infection testing and treatment, medical care from advanced practice providers supervised by physicians, and an on-site laboratory and pharmacy.

### Ethical Considerations

Ethics approval was granted by the primary institution from which data were collected to Meharry Medical College (21-07-1105), with review also conducted by the University of Memphis (PRO-FY2022-201). All participants completed an informed consent process prior to participating in the study; these were administered verbally, with written consent provided. All identifying information was kept confidential, with only the study principal investigator and two coders having identifiable information. All identifiers were removed, including connections of any quotes with participant demographic information that could be discernible. All personnel participating in interviews received a US $50 gift card as compensation, and all steering committee members received a US $50 gift card as compensation for attending the 1.5-hour meeting.

### Study Approach

Overall, this research is a product of a pragmatic approach in which research is focused on practical understandings of concrete, real-world issues [26]. To advance a pragmatic approach, we applied a constructivist stance to collecting data through multiple methods and from multiple sources, interpreting their meaning, and reporting key findings and implications. A constructivist stance emphasizes the importance of individuals constructing their own understanding and interpretation of the world through personal experience and reflection and lends itself well to the objectives of research co-production, where ongoing, collaborative input shapes the research design and execution [26-29].

We used a multiple-methods design to complement the constructivist stance, recognizing that this combination would best facilitate the integration of varied perspectives within our team [26]. Mixed methods designs support the generation of stronger inferences when compared with a single medium of data collection by enabling the capturing of a more holistic and richer illustration of complex phenomena [30]. Moreover, this design is recommended for research that is community-engaged [31]; it aligns with the iterative nature of co-production and is well-suited for TIC as a paradigm, indicating the need to include voices from multiple platforms in a system and through various mediums to enhance inclusivity. An exploratory sequential mixed methods design was applied for data collection: first, collecting qualitative data through in-depth interviews; second, administering a brief quantitative and qualitative survey instrument; and then, synthesizing these findings. This approach facilitated the triangulation of multiple lines of evidence, multiple sources of insight, and an integrated analysis and discussion.

The current formative research presents one part of an ongoing effort to collaboratively implement TIC in the youth-focused HIV clinic. Previous findings have informed the current stage of research, and current findings will be applied to direct and tailor future TIC implementation specific to the setting. The first phase of the overall study was conducted during the first year of the project (2021) and has been published elsewhere [32]. This phase marked the inception of the project and was initiated by the focal clinic’s request for technical assistance with TIC implementation from the study team. During that phase, the study team primarily focused on soliciting or integrating local feedback on grant objectives and research questions via an established local community workgroup, Connect 2 Protect, and with internal and external local champions. These efforts were followed by information-sharing and rapport-building activities, including the study team presenting on TIC to clinic staff, as well as the institution as a whole, via a hospital-wide Grand Rounds didactic and the construction of a process map of clinic services. The process map was generated through discussion groups with personnel and a patient peer representative group; those findings served to construct a joint understanding of standard care and available resources, as well as limitations and the need to develop protocols relative to TIC. Findings from the process map were used to update a pre-existing interview guide that had also been developed through community workgroup discussions for TIC in HIV care spaces [33,34].

### Current Data Collection

The next phase of activities for this study occurred in the spring of 2022 and primarily centered on engaging clinic personnel to purposively participate in one-on-one key informant interviews to assess barriers and facilitators to TIC implementation. Inclusion criteria included all patient-facing clinic personnel. Once identified as clinic personnel by the site principal investigator, eligible individuals were invited via email to participate. All individuals who volunteered to participate were invited for an interview and asked to choose if they preferred a web-based (Zoom; Zoom Video Communications Inc) or an in-person platform. The 30-minute to 1-hour interviews were conducted by two master’s-level public health students operating under the supervision of a PhD-level investigator (LCP), with the interviewers having no previous relationship with personnel of the clinic. Both interviewers received training on the topics of HIV and TIC prior to conducting the interviews. Interviews were then audio recorded and transcribed verbatim.

During the final phase of this study (summer 2023), a steering committee of approximately 18 clinic personnel was convened to begin designing a plan for collaborative TIC implementation. The steering committee composition was built primarily from clinic personnel who volunteered to participate and was comprised of providers, researchers, and administrators and additionally included the project principal investigator and several topic experts specializing in TIC and co-production, with the intent to invite patients onto the committee after discussions within the group of best practices to promote patient emotional safety. The steering committee met three times and was then invited to participate in a web-based survey to elicit a contextual understanding of baseline levels of the co-production environment using the Research Quality Plus for Co-Production (RQ+4 Co-Pro) framework [35]. Participants received US $50 gift cards for attending steering committee meetings. Multimedia Appendix 1 illustrates how each phase of the research thus far has iteratively built on a collaborative approach for TIC implementation.

### Statistical Data Analysis

This paper focuses on findings from a mixed methods designed study (Multimedia Appendix 1), which collectively sought to (1) explore implementation determinants and (2) conduct a dynamic evaluation. First, primary data from interviews were analyzed using the Consolidated Framework for Implementation Research (CFIR) 2.0 [36] to deductively code qualitative data, allowing CFIR constructs and domains to emerge de novo in our analysis. Four cycles of coding were conducted by researchers who were external to the clinic environment, with all three being female, with a mixed racial composition, including AJA and JLS for all cycles, and LLB was involved in the first and final cycle. First-cycle coding began with the three coders coding the first transcript together, followed by the two coders (JLS and AJA) independently coding each transcript by reading transcripts as PDFs and documenting notes into Excel (Microsoft Corp) by CFIR domains. Spreadsheets were organized first by CFIR domains, with columns listed for a summary of each domain, themes presenting as barriers with sample quotes, and themes presenting as facilitators with sample quotes. CFIR subdomains and constructs were then listed within each CFIR domain, with the same information for each subdomain and construct. Upon review of these results, all coders felt data saturation had been reached and no further interviews were required. Second-cycle coding occurred with the two reviewers verbally discussing the independently-developed results to begin developing a codebook based on presenting CFIR themes and revising contextually-defined definitions and summaries of each construct. Third-cycle coding entailed reaching consensus on which quotes belonged in which CFIR domains and constructs (eg, differentiating similar themes by grouping together ideas into one theme most befitting). A draft of these findings was then developed as a narrative Word (Microsoft Corp) document, with researcher LLB reviewing themes and discussing discrepancies with JLS and AJA to reach consensus. Results from Phase II were then reviewed with the steering committee to discuss the meaningfulness of findings and a general plan for coproducing the implementation of TIC in light of the findings.

Results from interviews were used to adapt the pre-existing framework, RQ+4 Co-Pro, which uses both quantitative and qualitative survey responses [37].

Adaptations focused on ensuring the language was appropriate to the current setting and study, relative to TIC in the HIV clinic, with personnel who were generally unfamiliar with the concept of co-production and included insights from the first phase findings [32]. Three authors (LLB, RKDM, and JLS) worked together to adapt the tool. The RQ+4 Co-Pro framework was designed to explore the local context for co-production. The RQ+4 Co-Pro contextual factors are: (1) Knowledge Use Environment, measuring appetite for research-generated knowledge and the ability to use evidence-based approaches to inform practices and policies (restrictive vs empowering); (2) Research Environment, measuring the extent to which an environment is supportive of co-production as an effective and legitimate approach to research (restrictive vs empowering); and (3) Capacities for co-production, measuring the extent to which the environment is devoting time and resources to build the capacity of researchers and users or beneficiaries to engage in co-production (no focus vs strong focus). Each of the three contextual factors includes a 4-point rubric to enable a quantitative rating and an open-ended short response to allow examples and qualitative descriptions.

To analyze RQ+4 Co-Pro results, we applied content analysis, using the RQ+4 Co-Pro framework to organize responses, with themes reviewed between three coders (LLB, JLS, and RKDM). Descriptive statistics were used to analyze quantitative response data for each of the three RQ+4 Co-Pro rubrics. Following the analysis of interviews using CFIR, a final cycle of coding was conducted by LLB and RKDM to synthesize findings from CFIR with those of RQ+4 Co-Pro. To synthesize data, we followed a deliberative dialog approach [38] to collectively interpret how findings from each phase of the research contributed to an overall understanding of the potential utility of using a collaborative approach as a trauma-informed research strategy in the clinic. Deliberative dialog is an approach that allows multiple parties to consider relevant information from multiple points of view [38]. Our process involved two team members (LLB and RKDM) undertaking independent analysis, followed by shared reflection to identify any points of divergence or similarity. These points were explored between the two team members, and consensus was then achieved through review by the full research team. Because the CFIR instrument is not intended for use at the patient level, patient interviews required a separate analytical framework for interpretation and will be presented in a forthcoming study.

## Results

### Sample

Interviews were conducted (March-August 2022) with 20 (57%) clinic personnel, of which 18 (90%) identified as female, one male (1%), and one (1%) nonbinary; 8 (40%) participants identified as Black, 8 (40%) as White, 2 (10%) as Asian, and 1 (1%) as Native American/Hawaiian; median age was 48 (IQR 42-54) years; median time in the position was 14 IQR (1-20) years. Positions included mental health practitioners (eg, social workers, chaplains, and psychologists), physicians, advanced practice providers (eg, physician assistants and nurse practitioners), nurses, clinical researchers, patient representatives, and administrators. RQ+4 Co-Pro surveys were conducted with a total of 9 (50%) steering committee members in July 2023.

### Interview Results

Four CFIR domains emerged as potential implementation determinants, with various CFIR constructs presenting as relevant for each domain: (1) Outer Setting, (2) Inner Setting, (3) Individuals, and (4) Implementation Processes. Findings are organized by domain and constructs below with exemplary quotes. Definitions and other quotes are provided in Multimedia Appendix 2.

### Outer Setting

The Critical incidents construct indicated that personnel overall felt the COVID-19 pandemic presented opportunities for strengthening engagement between personnel via new web-based group meetings during a difficult time (eg, hospital-wide groups convened to discuss national and local reports of police brutality). However, personnel also discussed the perception that COVID-19 presented challenges for patient engagement, coupled with a greater need for patient support, with an indication that such challenges potentially moved them further from a TIC paradigm. Additional relevant incidents included the clinic’s move to a new building and a new electronic medical record, with both identified as slowing down efforts to work toward TIC implementation. The move to the new building presented both opportunities for the HIV clinic to be more integrated with the overall hospital (ie, potentially destigmatizing HIV), as well as the concern that patients may be exposed to personnel, patients, or families with less experience interacting with persons with HIV or identifying as Lesbian, Gay, Bisexual, Transgender, Queer, Intersex (LGBTQI).

*I think the pandemic has definitely impacted us and caused more trauma. So, I think we’re definitely all very intimate with it. I think everybody’s experienced COVID trauma, whether you’ve had a personal experience or just the way that the world is. So, I think it maybe might open us up to talk [… ] But I also think there’s a barrier with time still*.

[Transcript 9]

Local attitudes indicated there was a perceived disconnect between the larger institution and the HIV clinic, with observations that patients with HIV do not receive the same services or amenities as patients in other hospital clinics. One person explained that this was perceived to contribute to stigma not only among patients but also among personnel of the HIV clinic.

*I don’t feel a larger embrace of [the] hospital for our patient population. We tend to be left out of events, there have been times we haven’t even heard of events or giveaways. It’s like we have been excluded for a long time.*

[Transcript 1]

Themes in Partnerships and Connections indicated that personnel felt there was hospital-wide support for multidisciplinary team meetings for providers to discuss difficult cases, and the option for research teams to engage a community advisory board (CAB) for feedback on research. While patients’ needs were seen as addressed through advanced referral networks for external mental health services, more community networks may be needed to help patients with basic needs (eg, housing).

*So that [CAB] feedback is very, very important, and I think that’s one of the things that we rely on when we’re looking at how we’re going to change things or if we are thinking of implementing new things.*

[Transcript 10]

Themes in Policies and Regulations indicated that the hospital had several policies and initiatives aligned with the HIV clinic’s human equity focus, including employee resource groups with an LGBTQI focus, family advisory groups, and a diversity, equity, and inclusion (DEI) curriculum for personnel. Moreover, it was elucidated that personnel felt the best way to implement sustainable change is by anchoring efforts into hospital-wide policies. Prioritization of confidentiality and privacy was highly valued throughout the institution, with attention to policies that promote patient choice and autonomy regarding information-sharing. While there were institutional mechanisms discussed for obtaining patient feedback and experiences via an annual patient survey and CABs or advisory groups, these mechanisms were seen as needing to be revamped, with attention to reducing power differentials within group settings to increase transparency and dissemination of patient survey findings.

*Once you make it a structural change, then everybody’s on board and responsible for it, and so those tend to be the changes that are most impactful and last.*

[Transcript 1]

### Inner Setting

The Physical Infrastructure care space was perceived as generally safe and inviting for patients (eg, gender-neutral bathrooms, privacy, closed doors, affirming posters, and well-lit rooms with televisions, games, blankets, and snacks); however, some expressed that the space could be more adolescent-friendly, and the new space (ie, integrated within the hospital) may have reduced privacy and less exam rooms.

*Now we are in a different clinic, and it’s a much newer building. However, you’re kind of wide [… ] out and in the open; you are mingled in with the rest of individuals in the hospital.*

[Transcript 17]

The Information, Technology, and Work Infrastructure was perceived as well-developed. Responsibilities and chain of command were seen as clearly demarcated, with various types of mental health assessments conducted by personnel from different disciplines, and comprehensive care provided by an on-site pharmacy (eg, adherence counseling). Conversely, a need was raised to make screenings more comprehensive and deduplicated to reduce repeat suicide assessments and patient fatigue, and some felt that there was a lack of understanding of duties and activities among personnel of different positions.

*I feel like some additional questions could be asked specifically about sexual traumas and things of that nature. Because I don’t recall asking, we ask about domestic violence, but not anything specifically about sexual.*

[Transcript 19]

Communication and Related Connectedness indicated that personnel perceived clinic practices generally as promoting inclusivity and safety among patients, and a strong sense of team integration allowed patients to be well supported. While most staff reported feeling prepared to handle patient trauma due to on-the-job experience, there was an expressed need for patients to be provided more options regarding the order of clinic services received (eg, blood draws at the start of appointments vs later in appointments), and a need for more frequent staff meetings to improve communication about patients’ needs, with specific attention to meeting the mental health demands stemming from trauma.

*We have such [… ] great communication with the multidisciplinary team, that we’re all on board of what is going on with patient[s]. What have they shared*.

[Transcript 5]

Human-equity centeredness themes emerged relative to how the clinic fosters respect for patient cultural differences as seen with patient-affirming policies (eg, use of preferred names and patient-identified pronouns). Further, clinic personnel expressed having a high level of care and compassion, with an accentuated respect for providing care equitably and working to destigmatize HIV hospital-wide. Despite this, there was a stated need to train personnel on covert HIV stigma and unconscious bias and to expand the personnel understanding of racial trauma.

*We always let them know that they can be who they want to be regardless of how they were born or how they identify. We will respect whatever decisions they make [… ] or [… ] for the person that they are.*

[Transcript 19]

The clinic environment was perceived to be recipient-, deliverer-, and learning-centered with shared values, beliefs, and norms around caring, supporting, and addressing the needs and welfare of recipients and deliverers, as well as the prioritization of psychological safety, continual improvement, and the use of data to inform practice. Personnel were reportedly attuned to patient experiences of trauma and HIV-related stigma, as well as a perceived stigma against providers working in the field of HIV. Generally, personnel felt that work-life balance was promoted in the setting. On the one hand, personnel were seen as empowering patients, as patients are encouraged to communicate their needs and concerns and see themselves as equal players in health care decisions. On the other hand, some providers were seen as having limited flexibility for patients, prioritizing their own work schedule over the patients’ when booking appointments. More peer-to-peer support opportunities for patients were an identified need to enhance patient care, and personnel identified that a clinic-level patient needs assessment should be conducted to elicit patient clinic experiences related to safety and culture. Finally, the clinic was seen as a space in which there is a positive learning climate where new practices are relatively easily adopted (eg, past studies evaluating the impact of staff being trained in motivational interviewing and staff wearing pronoun buttons). Greater efforts are reportedly needed in the clinic to reduce burnout and provide more opportunities for staff promotion or appreciation, but some identified more tenured staff appear resistant to innovations.

*This will just go back to the inability for people to be adaptable to change. So being stuck in those cultural norms that they’ve already learned, they’ve already practiced and not being adaptable to learning new things.*

[Transcript 12]

A strong tension for change was observed. Personnel stated that TIC implementation was a priority (eg, expressed need for comprehensive and routine patient trauma assessments) but identified time constraints and barriers to providing more robust mental health services (eg, high levels of staff turnover or burnout, regular exposure to patient trauma, need for inclusive policies hospital-wide to reduce patient stigma, or likelihood for retraumatizing patients).

*… I think primarily it’s in staff morale, staff turnover, and then seeing compassion fatigue just in individual providers in stress and overwhelm and maybe even a lack of confidence in their ability to do the work that we know they know how to do.*

[Transcript 3]

TIC was stated by all personnel interviewed as highly compatible with the desired clinic culture, the need for training staff, and the need for expanding patient and provider mental health care. Staff reported being mindful of patient stigma and responsive to how trauma effects influence patient behaviors, medication adherence, and treatment outcomes. There was an expressed need to educate patients and nonclinical personnel on how trauma affects health and about trauma response. Finally, there was a stated need to regularly integrate patient feedback and preferences to improve clinic services.

*I think it [TIC] fits [at our clinic]. I think it’s important because if we don’t come from that lens, I think we’re not providing optimal services. So yeah, I think it’s extremely important* ... *Because the personnel in the [HIV] Clinic are very embracing of our patients and understand they*’*ve been exposed to a variety of circumstances.*

[Transcript 2]

Personnel stated that TIC implementation was seen as a priority by all clinic personnel, but had been temporarily paused during the relocation and adoption of the new electronic medical record system. Resources identified to further prioritize TIC implementation were time, space, money, tools, and staffing (ie, limited exam rooms for private discussions and overworked providers). Assessments were identified as needing to be reconsidered, including adverse childhood experiences, which were seen as not comprehensively capturing some social determinants of trauma (eg, racial trauma and housing instability), general psychosocial assessments, and suicide risk assessments. While direct service providers reported having access to trainings (eg, motivational interviewing, cultural humility, and implicit bias or diversity), there was a perceived need to ensure all staff, including front desk and administrative, have access to the same trainings. Enhancements to training, such as more videos and role-plays to help prepare for interactions with patients and trauma, were suggested. Notably, personnel who mentioned tangible barriers (eg, training needs and learning curve) were generally longer-term employees.

*If all staff [get] involved and [receive] more training, we could pick up on triggers better and be able to get the patient help or share what we saw or perceived. And maybe work more closely with the team that way, especially with the social worker or the psychologist.*

[Transcript 5]

### Individuals

Project role themes emerged relative to high-level leaders and innovation deliverers. While providers unanimously supported TIC implementation and universal patient trauma assessments, and clinic leaders expressed unequivocal support for TIC, clinic and hospital leadership were perceived as potential barriers if they do not more greatly prioritize staff wellness.

*I think our biggest hurdle would be leadership. I think if something is not in line with their vision on their time, then it won’t be a primary focus.*

[Transcript 17]

The project characteristics subdomain included individuals’ needs, capabilities, and motivations. Needs emerged relative to staff stress or burnout or turnover and the impact of providing trauma stewardship for patients. Facilitators, conversely, included that personnel generally felt supported by a nurturing team environment and (eg, staff-peer support program and clinician-focused presentations) wellness initiatives (eg, institution-wide mindfulness hour).

*Listening to people’s traumas so often can lead an employee to be overwhelmed. It can lead an employee to being in a space of hopelessness because there’s so much trauma and not enough response to that trauma...*

[Transcript 16]

Most reported feeling adequately prepared to handle patient trauma due to experience and team support, but in-person trainings (vs web-based or recorded trainings) were seen as a means for improving skills for nonclinical staff to assist with HIV disclosure and effectively responding to patient trauma. Finally, personnel expressed a commitment to providing optimal care and addressing their own personal trauma, privilege, and biases, but efforts to adopt innovations were sometimes stymied by feeling overworked and immersed in a culture where patients are prioritized over personnel well-being.

*Just high levels of stress* ... *We’re not in a great state right now in terms of emotional well-being of staff* ... *We need protected time specific to providing self-care. It needs to be a priority and it needs to be modeled as a priority by our staff, by our managers.*

[Transcript 5]

### Implementation Processes

Overall, the success of TIC implementation was perceived to rest on how well it could impact professional quality-of-life outcomes (ie, burnout) and clinic improvements in trauma screening and assessment protocols so that culturally responsive care is enhanced and more services are provided by people who mirror patients in race or ethnicity, age, gender identity, and lived experience.

*I feel like that can actually be a hindrance if you don’t have people who are relatable or look like the patient. How does that impact them to give you that information and make them feel safe?*

[Transcript 17]

### Interview and Survey Results Synthesized

Numeric results, summaries of qualitative responses, and sample quotes are provided below from the RQ+4 Co-Pro analysis for each of the framework’s three contextual factors. The tables presented inMultimediaAppendices 3^5^ present the synthesized results with the two frameworks, CFIR and RQ+4 Co-Pro, integrated together. Specifically, this table shows how CFIR findings reflected levels of evidence, support, and capacity for the collective co-production approach to research. We present a summary of the synthesis findings below, as well as in MultimediaAppendices 3^5^.

The contextual factor Knowledge Use Environment (eg, ability to use evidence-based approaches) was on a 4-point rubric (4=Empowering, 3=Supportive, 2=Unsupportive, and 1=Restrictive). The mean response was 3.6 (SD 0.70), indicating an environment highly conducive to using research results in decision-making. Qualitative responses indicated themes that appeared relative to current knowledge or research use, evidence as a priority, and efforts to adopt data-driven approaches (Multimedia Appendix 4). The hospital was perceived to be highly supportive of biomedical research by virtue of being a “research” hospital, with a culture for encouraging personnel to produce research and seek out innovations. However, one participant mentioned an example in which the HIV clinic faced a barrier to conducting research that other clinics in the hospital may not; this can affect the extent to which the clinic can engage in research. Finally, committee members reported approaching the process of research from a place of autodidactic curiosity and openness.

*I have learned through seasoned employees that surveys and research have contributed to programs that exist today like DEI - Diversity Equity and Inclusion.*

CFIR findings both affirmed these results and provided deeper context to demonstrate some potential challenges and facilitators that might present as teams consider research co-production approaches in the setting. Potential facilitators emerging from interviews focused on the clinic being seen as a learning-centered space, where new practices are relatively easily adopted, with personnel consistently referencing the use of standardized and validated instruments, evidence-based protocols, and various forms of hospital- and clinic-level efforts to improve outcomes via data-driven methods. While the RQ+4 Co-Pro items did not elicit barriers to the prioritization of evidence-based practices, CFIR themes indicated that while there are areas in which patient care services could be improved, some more tenured staff were perceived as resistant to innovations, and the perceived disconnect between the larger institution and HIV clinic could constrain collaborative research production.

 The contextual factor Research Environment (eg, level for which co-production is seen as effective and legitimate) was on a 4-point rubric (4=Empowering, 3=Supportive, 2=Unsupportive, and 1=Restrictive). The mean response was 3.1 (SD 0.31), indicating a supportive environment for research being cogenerated by personnel and patients. Qualitative responses indicated themes of examples of collaborative research in the setting, perceived value of collaborative approach, and the extent to which collaborative research approaches are used as the status quo (Multimedia Appendix 4). Findings suggested that research is done through a team approach. Members reported having collectively participated in many quality improvement projects to improve patient outcomes, in which all are treated as equal members of research teams; there are some leaders who encourage personnel to participate in conducting research, with the hopes of using results to reduce health disparities. The general belief expressed was that the collaborative approach aligned with the clinic and institutional culture. Other members felt that personnel had competing priorities, which slowed down research processes. Finally, one committee member indicated that they had no knowledge of co-production occurring in the environment.

*We all are able to propose potential changes in our practice and back it up with evidence*.

Some of the facilitators identified during interviews appeared to support findings from RQ+4 Co-Pro. Specifically, personnel indicated a shared value for seeking and integrating patient feedback to improve care, and evaluation approaches were discussed as embedded in clinic policies, procedures, and practices in which end users are perceived to contribute equally to research teams and actively contribute to change efforts. Conversely, potential barriers centered around perceived silos between positions and some staff feeling their skills are underused relative to research activities. Overall, personnel indicated novel approaches should be anchored into standard operating procedures to be sustainable.

Finally, the contextual factor Capacities for Co-production (eg, the extent to which research beneficiaries are trained on and have resources for co-production) was also assessed on a 4-point rubric (4=Strong focus, 3=Significant focus, 2=Minimal focus, and 1=No focus). The mean response was 3.1 (SD 0.22), indicating there was significant capacity for research co-production. Qualitative responses indicated themes included training and capacity-building, resources, and support (Multimedia Appendix 5). Responses suggested a range of perceptions—that the clinic was more engaged in co-production than the hospital overall, to the inclusion of an example project in which the hospital had applied similar methods to develop the institution’s DEI initiative. Some members warned there would be a need for more time and resources to make co-production a standard approach, including patient care coverage. Other members (including some in nonresearch roles) reported they were learning by doing, and “a clear plan is not in place but in progress.”

Interviews indicated that many aspects of the environment are currently facilitative of coproduced TIC, including team integration, camaraderie, and the stated prioritization of TIC, underscoring the potential collaborative participation in germane research activities. Potential barriers included a perceived lack of formal training in research activities for patients and personnel, coupled with the perception that there are fewer resources currently available for the HIV clinic, which could limit personnel capacity for focusing on efforts outside of clinical care.

## Discussion

### Principal Findings

Clinic culture presented as highly aligned with TIC values, with personnel expressing unanimous support for its adoption in the clinic. Although leaders expressed unequivocal support, some staff felt this support needed to be demonstrated through a greater focus on personnel wellness. Moreover, the potential for co-production also appeared strong but will require formal training and resource allocation to bring to action. We draw comparisons with other studies below for contextualization. Overall, findings from this study have clinical relevance for future implementation in the clinic but also progress knowledge in several meaningful ways. Generally, results contribute to the small but growing body of research on TIC with youth with HIV, as well as the potential for co-production as a useful strategy for TIC implementation.

Our findings reflect much of what has been observed across various settings, with studies consistently finding strong personnel support for TIC implementation and excitement for expanding trauma assessment and treatment protocols; this has been observed in other national pediatric care settings [39], HIV care organizations, and general health care spaces [15,33,40,41]. The current clinic’s equity-focused culture (ie, compatible with TIC) is consistent with a similar study from a Southern HIV clinic, with both indicating that despite a positive and compatible culture, TIC implementation will likely require capacity-building (eg, trainings and procedural integration into workflow) [24]. Findings from other youth-focused outpatient counseling centers echo this need, adding that trainings must be supplemented with attention to system-wide efforts (eg, interorganizational networks, staffing, and fidelity monitoring), and highlighting the shared need to advance systematic service user feedback and mobilize change-resistant personnel [40]. Concern for sustainability also appears to be shared across settings [15,40,41], with a need for interagency collaborations, support from leadership, and policy changes identified as key indicators of sustainability across youth-focused care settings [40]. Finally, our findings align with those of a large study across various pediatric mental health settings, indicating that internal champions are critical for supporting implementation of expanded trauma assessment and treatment protocols; however, this study additionally found that external change agents were also critical [39]. While the role of external change agents was not a theme in this study, this study is nested within a larger implementation project that relies on external change agents. Thus, clinic personnel could benefit from hearing how external change agents may act as facilitators.

Several barriers to TIC appear to be unique to this study, when compared with a systematic review of TIC across health care settings, in HIV clinics, and youth-focused organizations. Some of these included the perception that patients receive differing care or are stigmatized outside of the clinic, the lack of peer-to-peer support or platforms for soliciting or integrating patient preferences for care systematically, and the presence of repetitive suicide assessments, which could contribute to the likelihood of retraumatization. While we observed that more tenured staff were more likely to discuss tangible resource barriers, it was not discernible if these individuals had a more realistic view of challenges related to implementing innovations or if these perceived barriers could have been signs of perceived change resistance.

Regardless, most perceived barriers are consistent with other TIC literature, including among youth and in HIV care spaces. Some of these shared needs include enhancements in workforce development [17,40,42], staff wellness initiatives [17,40], trauma screening, assessment, referrals, and intervention [43], and the urgency for antiracism training for personnel [44]. McGeown et al [15] similarly found a need to direct attention to power dynamics as part of collective decision-making processes, and that more research skills, time, and resources are needed to implement TIC. Other collaborative trauma-informed research indicates that there are inherent power differentials in participatory research; academicians should stay cognizant to avoid having their own perspectives become the default and should solicit all members’ perspectives through formal feedback loops [45].

The general cohesiveness among clinic staff aligns with the TIC principle of Collaboration and differs from studies in which TIC has been scaled across macrolevel settings (eg, entire cities) [17,34]. However, cohesiveness may wane across larger spaces, as seen with the perceived disconnect between the HIV clinic and the wider institution. Methods for integrating initiatives are a focal point for future TIC research in this setting to ensure policy changes are most likely to be sustained. The hospital-wide DEI initiative presents as a prime area for initiating multilevel efforts to be able to provide all patient-facing staff with culturally relevant TIC trainings.

To our knowledge, findings mark the first depiction of co-production as an implementation strategy for trauma-informed HIV care. While others have suggested co-production may be applied to TIC implementation [15,46,47], no literature showcases this implementation strategy with TIC in the United States or with LGBTQI-specific populations, men, or persons with HIV [15,40]. Results suggest many aspects of the environment would be facilitative of collaborative TIC (eg, cohesive or welcoming culture, trialability, human-equity focus, positive learning environment, and prioritization of TIC). Identified needs for consideration in future interventions include enhanced engagement among hospital leaders to ensure all personnel have access to resources for and protected time to engage in TIC and research activities through policy changes for effort distribution and mandating training for all patient-facing staff. Clinic leadership could also support the development of more peer-to-peer patient support opportunities by collaborating with psychosocial providers and increasing attention to change-resistant staff during adoption through individual discussions.

The existence of patient- and community-advisory boards demonstrates there is a path for engaging end users for feedback, but personnel perceive that the activities of these committees could be improved by including more current patients. Efforts to engage active patients in current CABs are recommended. Findings indicated that the environment likely would be facilitative of research co-production; however, the inclusion of personnel outside of the HIV clinic (eg, transportation representatives and security guards) in initiatives may present challenges (ie, training nonclinic personnel in a trauma-informed approach). Engagement with leadership or management of personnel outside of the HIV clinic is recommended to create buy-in among direct reports and support intervention participation for all patient-facing staff in the hospital environment.

Responses suggested that the hospital and clinic environments, in which staff are empowered to participate in research, spur personnel to seek out innovations. Specifically, the team evaluation approach appears facilitative of co-production. However, competing priorities for nonresearch personnel present challenges to the collaborative approach, with an identified need to develop a clear path and capacities for co-production to succeed. Generally, results suggested that committee members supported the approach for TIC research, with an emphasized need to ensure time and resources are reserved for personnel engagement in activities.

### Limitations and Future Research

The current findings have several notable limitations. Application of purposive sampling may have introduced bias, as personnel most supportive of the study may have more readily volunteered to participate. Hence, findings may not be representative of all clinic personnel and cannot be generalized to represent personnel from HIV clinics outside of the current sample. Moreover, while data saturation was reached, the sample size for the RQ+4 Co-Pro component was small, precluding stratified results by demographics. Several of the RQ+4 Co-Pro items did not reap strong results, indicating full saturation may not have been reached. Patient-level data have not been presented here but will be presented separately in a follow-up report. Given that some of the RQ+4 Co-Pro items were not fully assessed, follow-up quantitative surveys should be conducted with a greater sample size of clinic and hospital personnel, which will serve to further validate qualitative results. While this study presents preliminary findings of our process to begin synthesizing a co-production framework (RQ+4 Co-Pro) with preimplementation stage research on TIC implementation (using CFIR), future research is needed to build on these methodological ambitions to further develop an integrated model. Such an effort will require a larger case study across multiple sites to extrapolate contextual factors enabling and constraining TIC research co-production and apply the Classical Test Theory (including principal components analysis) to test and refine psychometric properties of such a model.

### Conclusions

We conducted a mixed methods formative study to assess potential implementation determinants of coproduced TIC in a pediatric, youth, and young adult–focused HIV clinic in Memphis, Tennessee. Findings suggested that clinic personnel unanimously support TIC and there is an urgency for its implementation, but clinic and hospital leadership were perceived as potential barriers without more explicitly prioritizing staff wellness. Additionally, some staff shared concerns about change-resistant attitudes among some personnel, stating there would be a need for more peer-to-peer support opportunities for patients and greater efforts to anchor adoption into the larger hospital procedures, and strong support from multidisciplinary clinic leaders is perceived to be a vital factor in implementation. The clinic environment was also seen as an ideal space to conduct research, with a generally empowering milieu for co-production, but also, there was a stated need for a clear path to be formalized so that personnel have protected time for engagement and personnel wellness. Our results lead us to believe that TIC is likely to be an embraced evidence-based approach in the clinic, and co-production will likely be seen as a useful and fitting research strategy for TIC adoption among personnel of the sampled HIV clinic. However, follow-up research is needed to focus on integrating patient experiences and further test co-production (eg, for acceptability, appropriateness, and feasibility) as an implementation strategy for understanding and leveraging local contexts during TIC implementation. We aim to use results from this study to develop a site-tailored TIC implementation plan via research co-production so that personnel from the clinic remain equal contributors throughout the research process. Overall, both TIC and research co-production remain underused approaches in HIV care organizations, yet present as critical means for advancing national goals to end the HIV epidemic via collaborative, community-led solutions most likely to reduce health disparities. Findings from this study present potential implications for the multiple intersecting arenas of implementation science, adolescent health, and trauma research, all of which could be advanced from more greatly integrating either TIC or co-production into HIV care.

## Supplementary material

10.2196/66426 Multimedia Appendix 1 Co-production engagement in all phases of research.

10.2196/66426 Multimedia Appendix 2 Personnel-perceived barriers and facilitators to implementation of trauma-informed care in an adolescent-focused pediatric HIV clinic in Memphis, Tennessee

10.2196/66426 Multimedia Appendix 3 Knowledge use environment (eg, application of research).

10.2196/66426 Multimedia Appendix 4 Research environment (eg, value and actual use of collaborative approach).

10.2196/66426 Multimedia Appendix 5 Capacities for co-production (support for collaborative research on topic or in general).

## Abbreviations

CAB: community advisory board
CFIR: Consolidated Framework for Implementation Research
DEI: diversity, equity, and inclusion
LGBTQI: Lesbian, Gay, Bisexual, Transgender, Queer, Intersex
RQ+4 Co-Pro: Research Quality Plus for Co-Production
TIC: trauma-informed care
