# Supplementary material for: Exploring Co-production as an Implementation Strategy for Trauma-Informed Care in a Youth-Focused HIV Clinic in Memphis, Tennessee: Mixed Methods Research
Source: JMIR Form Res. 2025 Aug 21;9:e66426. doi: 10.2196/66426 (PMC12369914; doi:10.2196/66426)
Supplement: Multimedia Appendix 1 [file formative-v9-e66426-s001.docx]

|  | | | | |
| --- | --- | --- | --- | --- |
| Research design | Information sharing/ rapport building | Method development | Explore implementation determinants | *Dynamic  evaluation |
| Local community requests technical assistance specific to topic as locally relevant | Bi-directional education (e.g., from research team to hospital via  Grand Rounds)  Process map of clinic care via discussions with personnel and patient representatives | Interview guide developed by community workgroups and informed by process map results | Interviews—to assess perceived barriers and facilitators to adoption— conducted via external study personnel (to reduce bias in responses) and interpreted by external study personnel | Surveys to assess contextual factors influencing research engagement for institution |
| Community and internal champions engaged to inform grant-writing (i.e., research aims) |  |  | Interview results reviewed and interpreted by/ with steering committee; Results applied to develop focus group guide to guide to direct adaptation of evidence-based approach. |  |
| ***Note:*** Table depicts our total proposed process for conducting research co-production between 2021-2024 with personnel in a pediatric HIV clinic in the Southern United States, using an exploratory sequential mixed methods approach. *Dynamic evaluation is defined as an approach to research in which evaluation is on-going and iteratively conducted, with inferences drawn from the gestalt of a program of inquiry, to: 1) to understand whether innovation is creating impact, and 2) how engagement with the research is contributing to that impact. | | | | |
