# Supplementary material for: Exploring Co-production as an Implementation Strategy for Trauma-Informed Care in a Youth-Focused HIV Clinic in Memphis, Tennessee: Mixed Methods Research
Source: JMIR Form Res. 2025 Aug 21;9:e66426. doi: 10.2196/66426 (PMC12369914; doi:10.2196/66426)
Supplement: Multimedia Appendix 2 [file formative-v9-e66426-s002.docx]

|  | | |
| --- | --- | --- |
| *Domains & constructs* | *Sample quotes* | *Applicable TIC principles & implementation domains evident in quote* |
| ***Outer Setting domain*** | | |
| Critical incidents | *"we have competing priorities right now, pretty big ones”* (Transcript 3) | Collaboration needed between clinic and institution to reduce competing priorities (*Cross-sector Collaboration,* *Policy, Collaboration & Empowerment*). |
| Local Attitudes | *"sometimes it feels like we're the secret.”* (Transcript 1) | Perceived stigma for patients and care outside of the clinic, underscoring need for workforce development (*Cultural Responsiveness*, *Training and Workforce Development, Cross-sector Collaboration*). |
| Partnerships and connections | *"…the institution has a lot of support. […] a wellness center and a resilient program. […] support people who you can call if you're having a specific bad day […] We have mental health resources […] grand rounds where there are different topics [etc]* (Transcript 6) | Some institutional-level resources available for staff wellness promotion (*Safety* and *Cross-sector Collaboration*). |
| Policies and regulations | *"several of our patients have warrants. [It] is pretty intimidating when patients might use inappropriate anger or language in public areas, I've told staff to call myself or the social worker first don't call security, somebody in uniform...I'm not sure that I would be able to control or manage in the larger hospital if our patients acted out or used inappropriate language..."* (Transcript 4) | Need for training personnel outside of clinic (*Safety, Training and Workforce Development, Policy*, and *Physical Environment of the Organization*). |
| ***Inner Setting domain*** | | |
| Structural characteristics | *“We would like to learn if there are things we are not doing that we can incorporate in our clinical practice. […] What would be agnostic of role everyone should be doing?”* (Transcript 7) | Clinic seen as learning- and patient- centered (e.g., personnel can trial new approaches) but increases needed in patient trauma services and to obtain/ respond to feedback (*Empowerment, Voice, & Choice, Safety,* *Collaboration & Empowerment,* *Screening, Assessment, and Treatment,* *Quality Assurance; Evaluation).* |
| *Culture: Human Equity centered* | *“We may be showing, expressing stigma, even if that's not your intent."* (Transcript 7) | Identified need for further training to reduce stigma (*Cultural Responsiveness, Safety,* *Training and Workforce Development, Governance and Leadership*)*.* |
| Relational connections | *"I see a fair share of [providers] that have been practicing for long periods of time, but they aren't necessarily adaptable to change […] stuck in the ways that they were taught and practiced, as opposed to learning the recent innovations..."* (Transcript 12) | Despite referenced culture of open and honest communication, some staff may be change resistant, underscoring need for policy updates for feasibility and sustainability (*Engagement & Involvement, Progress Monitoring, Quality Assurance*). |
| Tension for change | *"* *Sometimes it can get to be overwhelming, and you don't have the luxury of just calling in and saying, I'm not coming today, [...] I'm exhausted. […] while you are in there dealing with other people's issues, it can become overwhelming. […] But you still have to come back and pick up.” (Transcript 8)* | Need to focus on reducing provider burden by building in supports and training (*Safety*, *Training and Workforce Development, Policy, Engagement and Involvement*). |
| Compatibility | *"I've got [patients…] who have been raped multiple times. […] And so they've been re-traumatized over and over […] again. And if we don't deal with the underlying issue […], they can't move forward.”* (Transcript 2) | Clinic staff have a shared recognition of patient trauma/ resilience and identified need to enhance trauma services (*Empowerment, Voice, and Choice,* *Screening, Assessment, and Treatment services*). |
| Relative priority | *“…assure that we're using tools that are empirically supported […] we'd have to have a process for response to those...we do screen for multiple different areas, but we don't do a comprehensive screening for trauma and effects right now."* (Transcript 3) | Need to enhance trauma services/ treatment (*Policy, Screening, Assessment, and Treatment Services*). |
| Available resources | *"I think that's [assessments] really key because if you don't know what you're dealing with, then you can't really work with a patient […] if we don't work on the issue initially, it will affect you long-term."* (Transcript 2) | Need to enhance trauma services/ treatment  (*Policy, Screening, Assessment, and Treatment Services*). |
| Access to knowledge and information | *"The hardships that many of our patients struggle with, that was never on my radar before working with our patient population. I had to learn a lot about HIV and a lot about stigma. […] I just thought they were just all in really bad moods […] I didn't realize how much they were struggling with, and once I did, it made things so much clearer for me […] to extend compassion and to not take them being standoffish so personally. But the trainings kind of help with that […] gives me an idea of what's going on, and what to be mindful of when I'm in those interactions that are a little more challenging." (Transcript 10)* | Widespread access to TIC education and resources needed (*Cross-sector Collaboration*; *Training and Workforce Development; Policy).* |
| ***Individuals domain*** | | |
| High-level leaders: Roles | *"We need administrative support, and it has to be a priority I guess, from an institutional level to say here are these resources to implement this screening and everybody needs to know this is important […] buy-in is really big and then also communication of that down the chain."* (Transcript 5) | Enhancements and championship needed for trauma responsive services and improved impact of provider training (*Governance; Leadership; Workforce Development and Training).* |
| Innovation deliverers | *"I think that they really need to bring in more of our research nurse staffing because I think we have the skills and the passion behind it to learn and do more in this area. […] It doesn't have to be this siloed situation."* (Transcript 9) | Desire to be more meaningfully engaged in patient care (*Empowerment, Voice, and Choice,* *Engagement and Involvement;* *Screening, Assessment and Treatment).* |
| Characteristics: Need | *"It [trauma] touches us daily. It impacts me with the way I live my life and the way I treat people." "...burnout is very, very, very big. Especially when you work in such a giving profession."* (Transcript 8) | Patient trauma is an identified occupational hazard, and greater support is needed to protect against the cost of caring (*Financing, Leadership, Safety).* |
| ***Implementation Processes*** | | |
| Champions | *"I don't think [TIC training] should be optional. I think that we need training from the doctor down to the front desk clerk."* (Transcript 1) | Universal training needed across sectors (*Training and Workforce Development; Cross-sector collaboration, Policy, Leadership*). |
| **Note:** Table depicts findings from qualitative interviews conducted in 2022 with personnel in a pediatric HIV clinic in the Southern United States, using an exploratory sequential mixed methods approach, with thematic analysis applied to analysis findings using the *Consolidated Framework for Implementation Research 2.0* and trauma-informed care principles from the Substance Abuse and Mental Health Services Administration’s Treatment Improvement Protocol 57 mapped onto findings. | | |
