# Supplementary material for: Exploring Co-production as an Implementation Strategy for Trauma-Informed Care in a Youth-Focused HIV Clinic in Memphis, Tennessee: Mixed Methods Research
Source: JMIR Form Res. 2025 Aug 21;9:e66426. doi: 10.2196/66426 (PMC12369914; doi:10.2196/66426)
Supplement: Multimedia Appendix 3 [file formative-v9-e66426-s003.docx]

|  | | | |
| --- | --- | --- | --- |
|  | | | |
| ***Integrated findings:*** Environment is learning-centered, where new and evidence-based practices are relatively easily adopted and improvement efforts typically apply data-driven methods, but some staff present as change resistant to innovations, and collaborative change efforts may be hampered by a disconnect between the institution and clinic. | | | |
| **Convergence and divergence between framework findings** | | | |
| Empowering - Supportive | | Unsupportive - Restrictive | |
| RQ+ 4 Co-Pro theme | CFIR findings | RQ+ 4 Co-Pro theme | CFIR findings |
| **Current research use:**  “[We are] a research institution, all ideas for new studies are evaluated by the team.  We have done many QI projects to reduce our no show rate, improve our transition to adult care; we all participated in MI training as part of research.” | | | |
| Hospital is a *research institution*, and the clinic has regular quality improvement projects to improve outcomes (e.g., Motivational Interviewing training to improve appointment adherence). | Clinic seen as a learning- centered space, where new practices are relatively easily adopted (e.g., staff wearing pronoun buttons). | Perception HIV clinic may not have same research opportunities (e.g., the HIV clinic may face unique barriers to conducting research). | Perceived disconnect between the larger institution and HIV clinic, with observations that patients with HIV do not receive the same services or amenities as patients in other hospital clinics. |
| ***Evidence* as a priority:**  “As a research institution, we are directed to look at evidence and/or pursue research to answer questions.” | | | |
| The culture and norms of the institution direct staff to apply evidence-based approaches. | Personnel reference use of standardized and validated instruments to measure patient outcomes. | Evidence not obtained | Greater efforts are reportedly needed to improve care, but some more experienced staff reportedly resistant to innovations. |
| **Efforts to adopt data-driven approaches:**  “We are curious and open to changing clinical guidelines and quality improvement.  If there is a question about clinical management, we will look up the latest research.” | | | |
| Openness to change and examples of context-informed efforts to improve outcomes. | Examples of hospital- and clinic- level efforts conducted to improve outcomes. | Evidence not obtained | Adoption of innovations potentially stymied by lack of protected time devoted to research. |
| **Note:** Table depicts a synthesis of findings from an exploratory sequential mixed methods approach in which qualitative interviews were conducted in 2022 with personnel in a pediatric HIV clinic in the Southern United States, followed by surveys conducted with a steering committee of personnel from the clinic in 2024. Interviews were analyzed using thematic analysis using the *Consolidated Framework for Implementation Research 2.0*, and surveys using Research Quality Plus for Co-Production (RQ+ 4 Co-Pro). A deliberative dialogue approach was followed to synthesize results from each framework. Example quotes are from the RQ+ 4 Co-Pro survey. | | | |
