# Supplementary material for: Exploring Co-production as an Implementation Strategy for Trauma-Informed Care in a Youth-Focused HIV Clinic in Memphis, Tennessee: Mixed Methods Research
Source: JMIR Form Res. 2025 Aug 21;9:e66426. doi: 10.2196/66426 (PMC12369914; doi:10.2196/66426)
Supplement: Multimedia Appendix 4 [file formative-v9-e66426-s004.docx]

|  | | | |
| --- | --- | --- | --- |
| ***Integrated findings:*** There are shared value for seeking/ integrating patient feedback to improve care, with evaluation approaches embedded and end-users perceived as contributors to change efforts, but the environment may be hampered by silos and under-utilization of staff skills; novel approaches should be anchored into institutional standard operating procedures. | | | |
| **Convergence and divergence between framework findings** | | | |
| Empowering - Supportive | | Unsupportive - Restrictive | |
| RQ+ 4 Co-Pro theme | CFIR findings | RQ+ 4 Co-Pro theme | CFIR findings |
| **Examples of collaborative research in setting:**  “The fact that this study is going on suggests the environment is at least supportive.” | | | |
| The current co-production study is evidence of collaborative research approach. | Staff perceived value of eliciting and integrating patient feedback to improve care. | Limited evidence happening in practice and evidence that some staff could not provide examples of a collaborative research approach in the setting. | Perception silos between positions have led to some personnel being under-utilized with research activities. |
| **Perceived value of collaborative approach:**  “As a research institution, we are directed to look at evidence and/or pursue research to answer questions. Participating in an HIV Stigma project is an example.” | | | |
| Belief co-production approach fits with hospital and HIV clinic focus, given the institutional history. | Team evaluation approach embedded in clinic policies, procedures, and practices in which end users are perceived to contribute equally to research teams and actively contribute to change efforts. | Limited evidence happening in practice but example by one staff that personnel incentives for promotion may not be currently well-aligned with the time and focus needed for co-production approach. | Perception novel approaches must be steeped into clinic model, with leaders embracing it as best methods for meeting patient goals, for it to become a standard approach. |
| **Extent to which collaborative research approaches are employed as status quo:**  “I have no knowledge of this actually happening.” | | | |
| Evidence of learning by doing. | Personnel perceived the current study to be a demonstration the clinic is invested in innovation solutions to improving care. | Perception patient engagement only available via community advisory board for research and not for clinic quality improvement. Research may not be translated to clinical care. | Need to more systematically elicit and integrate patient feedback to improve care. |
| **Note:** Table depicts a synthesis of findings from an exploratory sequential mixed methods approach in which qualitative interviews were conducted in 2022 with personnel in a pediatric HIV clinic in the Southern United States, followed by surveys conducted with a steering committee of personnel from the clinic in 2024. Interviews were analyzed using thematic analysis using the *Consolidated Framework for Implementation Research 2.0*, and surveys using Research Quality Plus for Co-Production (RQ+ 4 Co-Pro). A deliberative dialogue approach was followed to synthesize results from each framework. Example quotes are from the RQ+ 4 Co-Pro survey. | | | |
