# Supplementary material for: Exploring Co-production as an Implementation Strategy for Trauma-Informed Care in a Youth-Focused HIV Clinic in Memphis, Tennessee: Mixed Methods Research
Source: JMIR Form Res. 2025 Aug 21;9:e66426. doi: 10.2196/66426 (PMC12369914; doi:10.2196/66426)
Supplement: Multimedia Appendix 5 [file formative-v9-e66426-s005.docx]

|  | | | |
| --- | --- | --- | --- |
| ***Integrated findings:*** Environment currently facilitative of co-produced TIC (e.g., team integration, camaraderie, stated prioritization), underscoring potential collaborative participation, but there may be a lack of formal training in research activities, coupled with the perception there are less resources available for the clinic, limiting capacity. | | | |
| **Convergence and divergence between framework findings** | | | |
| Significant Focus – Strong Focus | | Minimal Focus - No Focus | |
| RQ+ 4 Co-Pro theme | CFIR findings | RQ+ 4 Co-Pro theme | CFIR findings |
| **Training and Capacity-Building:**  “Non researchers are learning the process of doing research and potentially creating new studies or projects…” | | | |
| Non-research personnel are learning about the relevance and usefulness of co-production via this project. | Many aspects of the environment are facilitative of collaborative TIC implementation, including team integration/ camaraderie, positive learning environment, and identified need for TIC, coupled with prioritization and compatibility of TIC. | Lack of formal training on co-production; clinic is learning co-production by doing but not via clear development mechanisms. | Lack of formal training for patients and personnel thwarts personnel perception of ability to respond to patient trauma. |
| **Resources:**  “I think we just need to formalize it.” | | | |
| Evidence of some informal training in biomedical research across positions via social learning environment. | Personnel perception TIC is a useful approach enhances potential for group to contribute to future co-production. | Perception collaborative approach is a natural fit with the HIV clinic but may differ from standard current approach of the institution. | Perceived need to systematize a co-production approach to utilize formal roles and procedures. |
| **Support:**  “…often folks feel pulled in a lot of directions so this can slow things down.” | | | |
| Perception co-production fits within the culture of the setting. | Providers unanimously supported TIC implementation and universal patient trauma assessments, underscoring the potential for personnel interest in participating in research activities. | Concern for time co-production will take compared with competing priority of patient care. | Perception there are less resources currently available for the HIV clinic, which could limit personnel capacity for focusing on efforts seen as outside of clinical care. |
| ***Note:*** Table depicts a synthesis of findings from an exploratory sequential mixed methods approach in which qualitative interviews were conducted in 2022 with personnel in a pediatric HIV clinic in the Southern United States, followed by surveys conducted with a steering committee of personnel from the clinic in 2024. Interviews were analyzed using thematic analysis using the *Consolidated Framework for Implementation Research 2.0*, and surveys using Research Quality Plus for Co-Production (RQ+ 4 Co-Pro). A deliberative dialogue approach was followed to synthesize results from each framework. Example quotes are from the RQ+ 4 Co-Pro survey. | | | |
